# Formal (4+1) Cycloaddition of Methylenecyclopropanes with 7-Aryl-1,3,5-cycloheptatrienes by Triple Gold(I) Catalysis**

**DOI:** 10.1002/anie.201404029

**Published:** 2014-06-04

**Authors:** Yahui Wang, Michael E Muratore, Zhouting Rong, Antonio M Echavarren

**Affiliations:** Institute of Chemical Research of Catalonia (ICIQ)Av. Països Catalans 16, 43007 Tarragona (Spain); Departament de Química Analítica i Química Orgànica, Universitat Rovira i VirgiliC/Marcel⋅li Domingo s/n, 43007 Tarragona (Spain)

**Keywords:** (4+1) cycloaddition, carbenes, cyclobutenes, gold catalysis, methylenecyclopropanes

## Abstract

7-Aryl-1,3,5-cycloheptatrienes react intermolecularly with methylenecyclopropanes in a triple gold(I)-catalyzed reaction to form cyclopentenes. The same formal (4+1) cycloaddition occurs with cyclobutenes. Other precursors of gold(I) carbenes can also be used as the C_1_ component of the cycloaddition.

Carbenes have been widely used as one-carbon synthon in organic synthesis, particularly in the context of cyclopropanation reactions.^1^ However, only a few (4+1) cycloadditions^2^ have been reported mainly with Fischer alkoxy(alkenyl)carbene complexes^3^ and dialkoxycarbenes.^2^, ^4^ To the best of our knowledge, there is no report on the (4+1) cycloaddition of aryl carbenes with 1,3-dienes, probably because of the known propensity of carbenes to give cyclopropanation products with 1,3-dienes.^5^ We postulated that due to their high strain and unique electronic properties, cyclobutenes^6^ could be used as synthetic equivalents of 1,3-dienes for the development of a formal (4+1) cycloaddition with metal carbenes.

We have recently found that 7-substituted 1,3,5-cycloheptatrienes **1** undergo gold(I)-catalyzed retro-Buchner reaction to form carbenes **2** (Scheme 1).^7^ Herein, we report a novel and potentially general formal (4+1) cycloaddition by reaction of **1** with methylenecyclopropanes **3**^8^ or cyclobutenes **4** to form cyclopentenes **5**. In this transformation, methylenecyclopropanes **3** undergo an isomerization to form cyclobutenes **4** similar to that catalyzed by platinum or palladium.^9^ Therefore, in the reaction between **1** and **3**, gold(I) plays a triple catalytic role, isomerizing **3** into **4** and, in parallel, generating gold(I) carbenes **2** from **1**, which cyclopropanate the cyclobutenes. Finally, gold(I) cleaves the internal C—C bond of the resulting bicyclo[2.1.0]pentanes to form the cyclopentenes. This reaction can be viewed as an insertion of one carbon into a double bond, a process that has only been achieved in rare cases with dihalocarbenes.^10^, ^11^

**Scheme 1:**
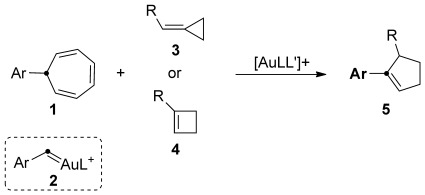
New strategy for the formal (4+1) cycloaddition.

Methylenecyclopropanes (MCPs) **3** can be readily prepared in one step by the Wittig olefination of carbonyl compounds with commercially available 3-bromo-triphenylphosphonium bromide. We first examined the reaction of phenylmethylenecyclopropane (**3 a**) with 7-naphthyl-cyclohepta-1,3,5-triene (**1 a**) in the presence of gold(I) complexes (Table 1). Using cationic [(JohnPhos)Au(MeCN)]SbF_6_ (**A**) in 1,2-dichloroethane at 120 °C, disubstituted cyclopentene **5 a** was isolated in 76 % yield (Table 1, entry 1). Other phosphine or N-heterocyclic carbene gold(I) complexes **B**–**E** gave lower yields (entries 2–5), whereas complexes **F** and **G** failed to promote this transformation, presumably due to their instability at the temperature required for the retro-Buchner reaction. The reaction also failed with silver(I), copper(II), and platinum(II) catalysts (entries 8–10).

**Table 1:** Gold(I)-catalyzed reaction of 7-(1-naphtyl)-1,3,5-cycloheptatriene (1a) with phenylmethylenecyclopropane (3a).^[a]^ 
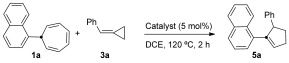

	Entry	Catalyst	Yield [%]^[b]^	
	1	**A**	81 (76)^[c]^	
	2	**B**	25	
	3	**C**	28	
	4	**D**	<5	
	5	**E**	47	
	6	**F**	–^[d]^	
	7	**G**	–^[d]^	
	8	**H**	–^[d]^	
	9	**I**	–^[d]^	
	10	**J**	–^[d]^	

[a] Reaction at 120 °C (0.2 m in 1,2-dichloroethane), 2 equiv of **3 a**, catalyst (5 mol %), 2 h. [b] Yields determined by ^1^H NMR spectroscopy using 1,4-diacetylbenzene as internal standard. [c] Yield of isolated product. [d] Not detected. 
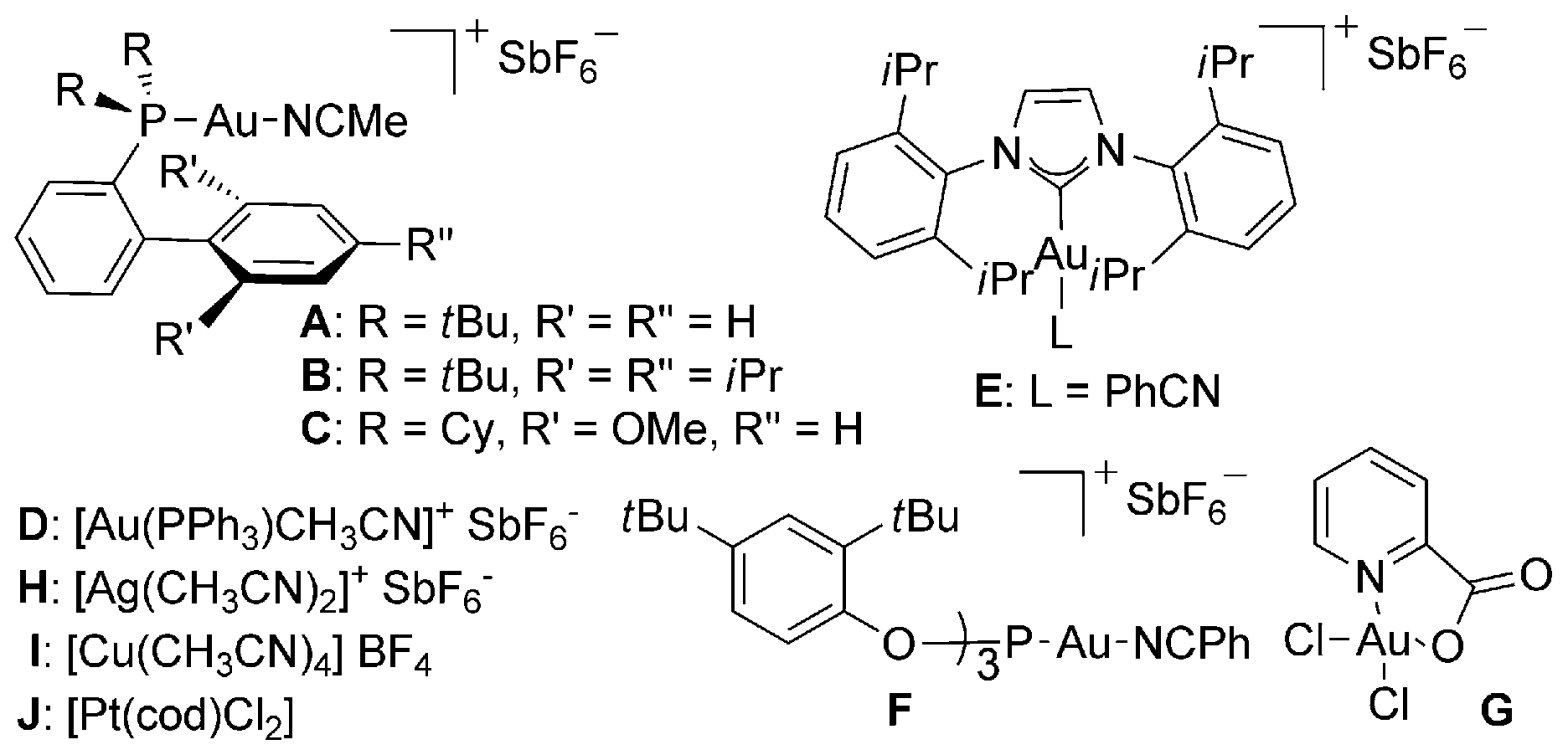

7-Aryl-cyclohepta-1,3,5-trienes containing groups with different electronic and steric effects at the *ortho*, *meta*, or *para* positions reacted with MCPs **3 a**–**h** to yield the (4+1) cycloadducts **5 b**–**m** (Table 2). The (4+1) cycloaddition proceeds satisfactorily with MCP bearing arenes with fluoro-, chloro-, and bromo-substituents. However, the reaction with *o*-bromophenylmethylenecyclopropane (**3 f**) led to cycloadduct **5 k** in lower yield. The structure of **5 k** was confirmed by X-ray diffraction (Figure 1).^12^ To demonstrate the synthetic utility of this method, cyclopentene **5 l** was prepared on a 500 mg scale using only 1 mol % gold catalyst **A** in 51 % yield after purification by column chromatography. Alkylmethylenecyclopropanes also reacted to give (4+1) cycloaddition products, although in this case the reactions led to mixtures of regioisomers **5 n/n′**–**5 p/p′**.

**Figure 1:**
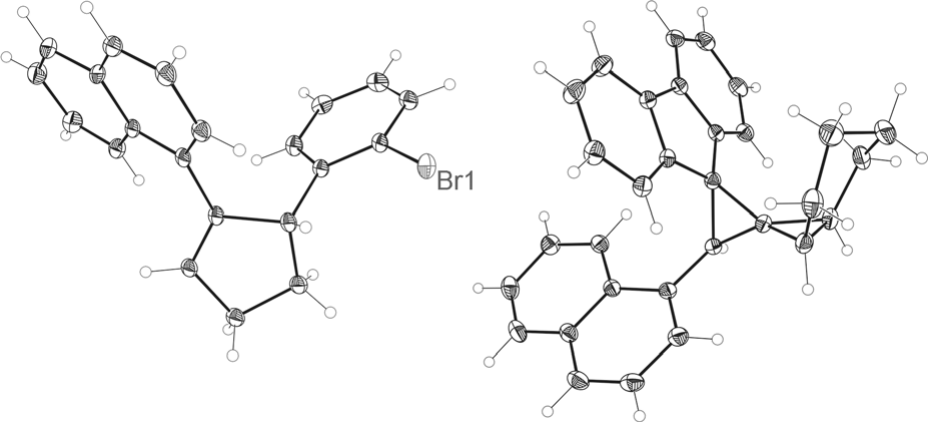
X-ray crystal structures of 5 k and 7.

**Table 2:** Scope of the formal (4+1) cycloaddition between cycloheptatrienes 1 and methylenecyclopropanes 3.^[a]^ 
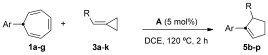

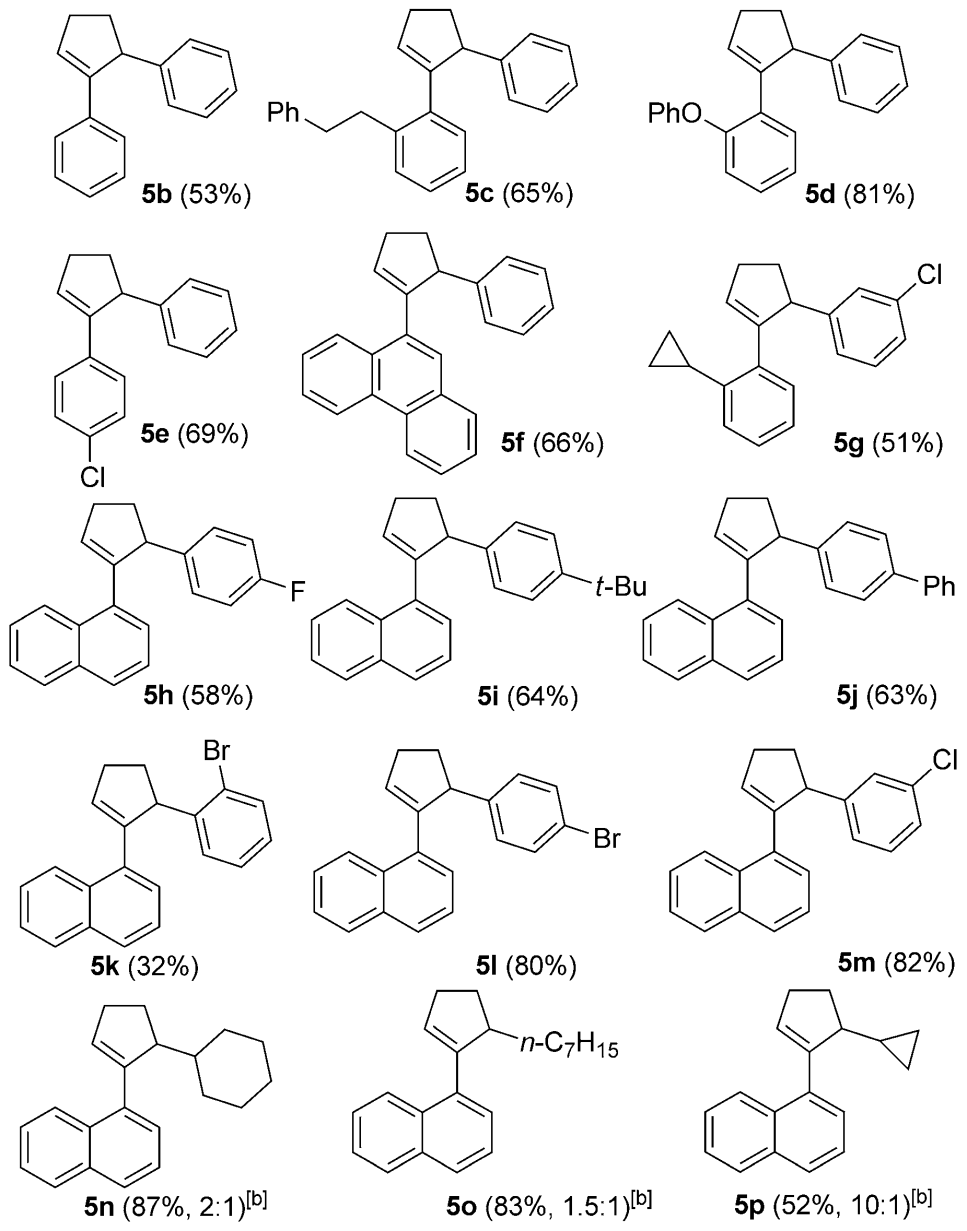

[a] Reaction at 120 °C, 0.2 m in 1,2-dichloroethane, 2 equiv of **3 a**–**k**, catalyst **A** (5 mol %), 2 h. Yields are for isolated products. [b] Reaction time=3 h. 3-Alkyl-3-arylcyclopent-1-enes **5′n**–**p** were also obtained as minor regioisomers.

Substrate **3 l** reacted intramolecularly using catalyst **E** to form 2,3-dihydro-1*H*-cyclopenta[*l*]phenanthrene (**5 q′**) by isomerization of the initially formed adduct **5 q** (Scheme 2). In addition, polyarene fragments can be obtained by photochemical cyclization. Thus, compound **5 f** can be transformed into a cyclopenta derivative of benzo[*g*]chrysene (**6**) by a one pot photo-induced isomerization/oxidative Mallory cyclization.^13^

**Scheme 2:**
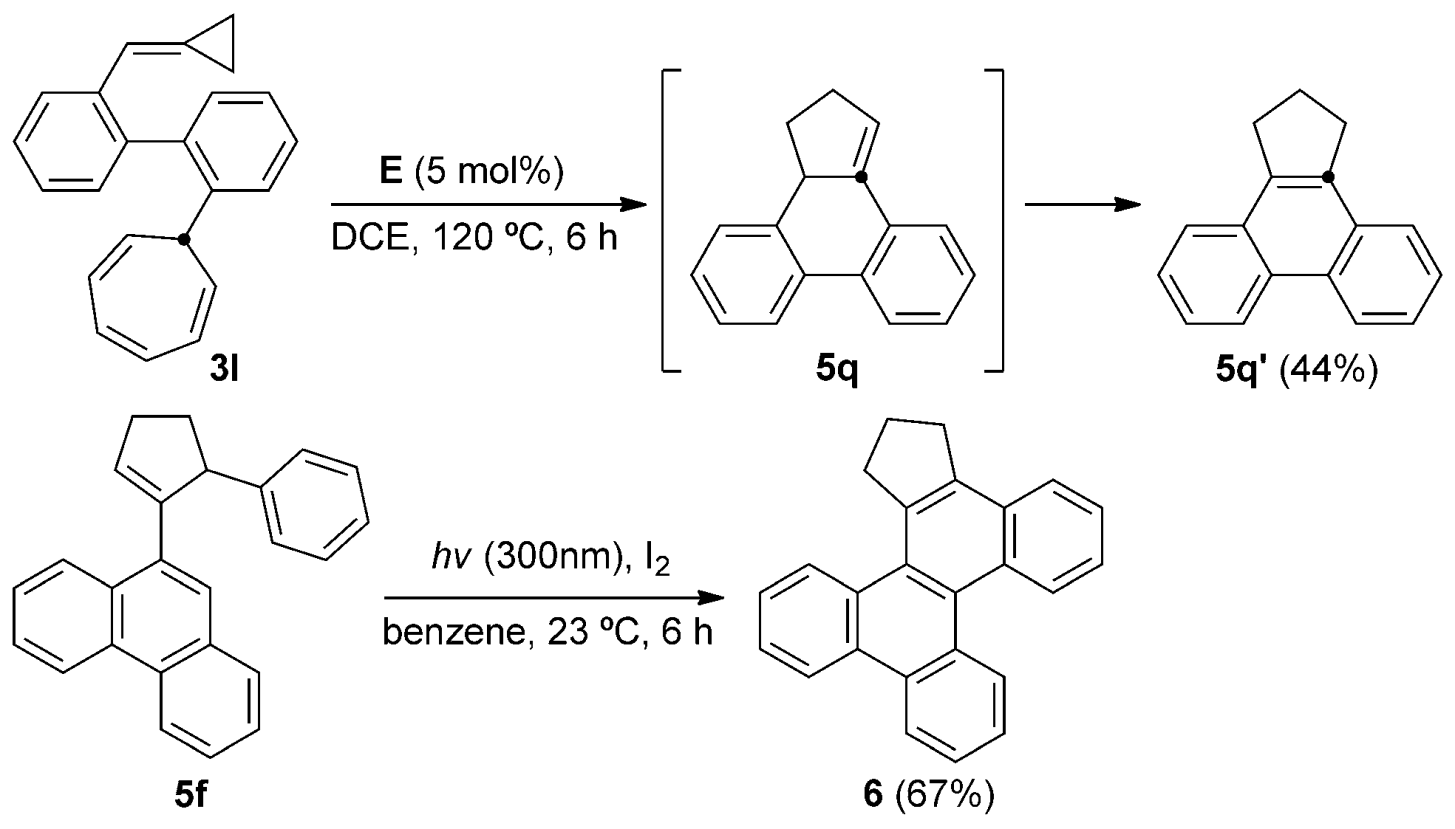
Intramolecular formal (4+1) cycloaddition and its application to the preparation of a polyarene fragment.

Tetrasubstituted MCP **3 m** reacted with **1 a** to give only the product of cyclopropanation **7** (Scheme 3 and Figure 1), whose structure was confirmed by X-ray diffraction (Figure 1).^12^ Given that **3 m** does not undergo ring-expansion, the isolation of spiro derivative **7** strongly suggests that the cyclopropanation of MCP is not the initial step in the formal (4+1) cycloaddition and that cyclobutenes are likely intermediates in this transformation.

**Scheme 3:**
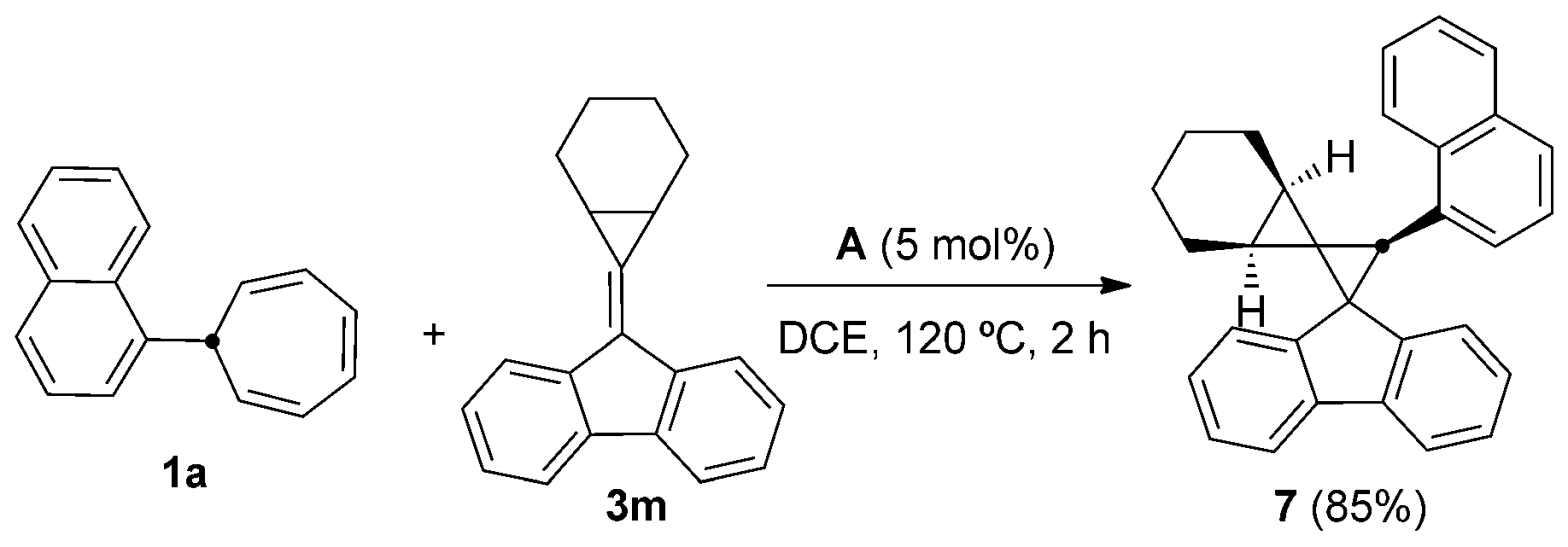
Probing the mechanism of the formal (4+1) cycloaddition.

To confirm the hypothesis that cyclobutenes are intermediates in the (4+1) reaction of MCP, we performed the reaction of **1 a** with cyclobutene **4 a**, which was isolated from the reaction mixture of **1 a** and **3 g**. Under identical conditions, cycloadduct **5 l** was isolated in 77 % yield. Trisubstituted cyclobutenes^14^ also took part in the (4+1) cycloaddition reaction to afford cyclopentenes **5 r**–**z** (Table 3).

**Table 3:** Scope of the formal (4+1) cycloaddition between cycloheptatrienes 1 and cyclobutenes 4.^[a]^ 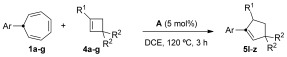

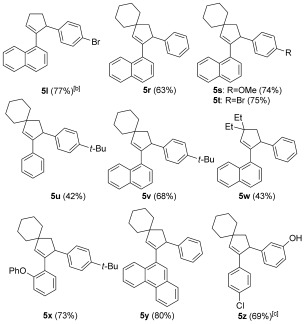

[a] Reaction at 120 °C, 0.2 m in 1,2-dichloroethane, 2 equiv of **4 a**–**g**, catalyst **A** (5 mol %), 3 h. Yields are for isolated adducts. [b] Cyclobutene **4 a** was isolated from the reaction mixture of **1 a** and **3 g**. [c] 2 Equiv of 7-(4-chlorophenyl)cyclohepta-1,3,5-triene were used.

Cyclobutenes also react with intermediate gold(I) carbenes generated by 1,2-acyloxy migration of propargylic acetates^15^ under mild conditions with catalyst **E** to give two separable isomers **5 aa**–**ac** and **5′aa**–**ac** in good overall yields (Scheme 4). By performing the reaction at room temperature at only 60 % conversion, bicyclo[2.1.0]pentane **10 a**^16^ could be isolated and then transformed cleanly into **5 aa** at 40 °C in the presence of gold(I) catalyst. The gold(I) carbene generated from phenyl diazomethane^17^–^20^ reacted similarly at room temperature with cyclobutene **4 c** to form the desired formal (4+1) product **5 ad**, along with **10 b**.^21^ This bicyclo[2.1.0]pentane was converted quantitatively into cyclopentene **5 ad** by warming at 60 °C in the presence of gold complex **A**.

**Scheme 4:**
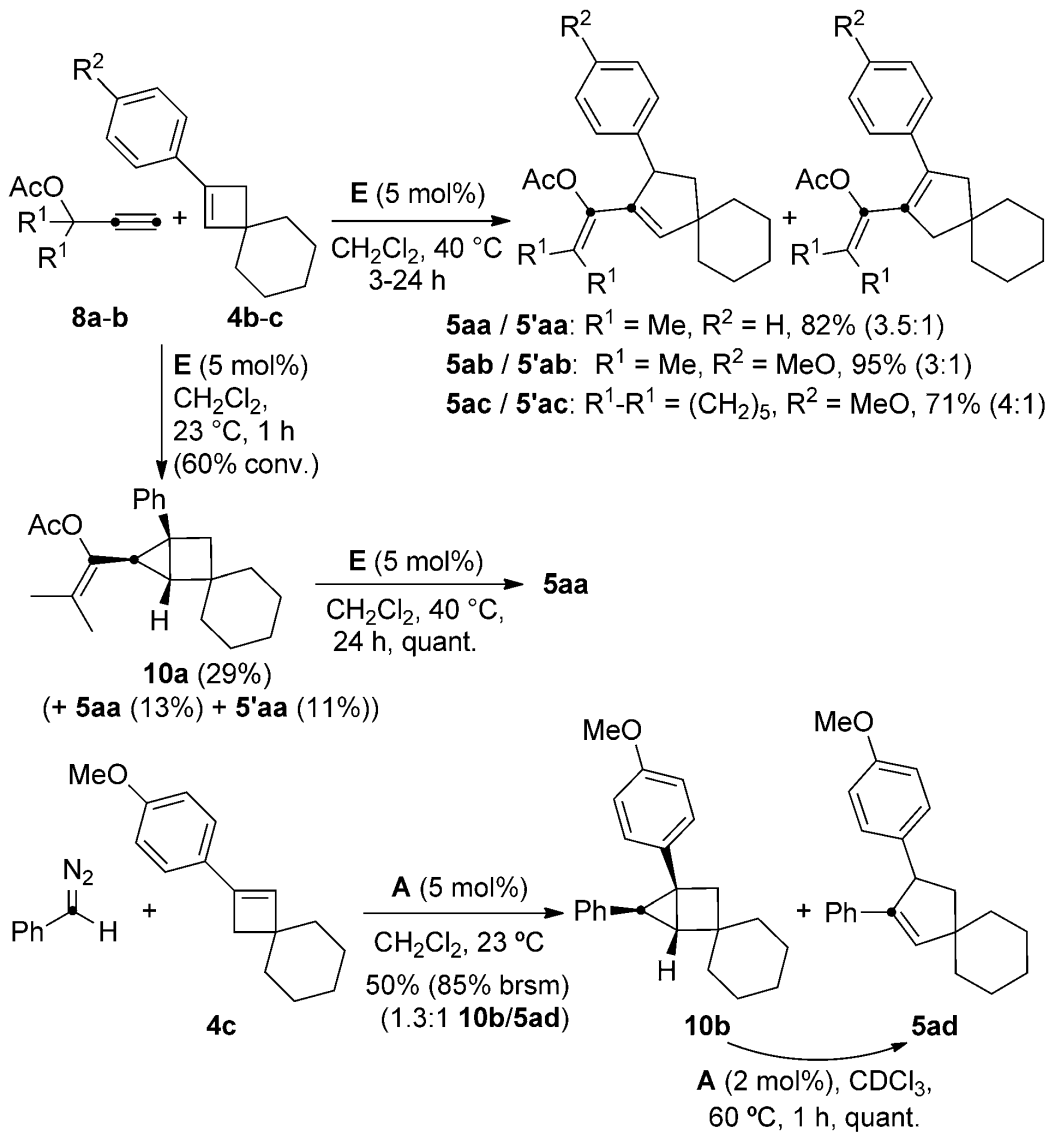
Formal (4+1) cycloaddition with various gold-(I) carbenes.

To shed additional light on the reaction mechanism, we performed the reaction of cycloheptatriene **1 a** with MCP [D_1_]-**3 a** in the presence of catalyst **A** (Scheme 5). In this experiment, [D_1_]-**5 a** was obtained with the deuterium label transferred completely to C-3.

**Scheme 5:**
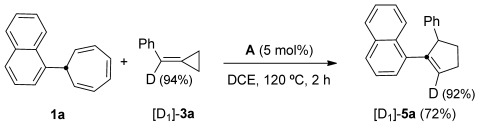
Deuterium labeling experiment to probe the mechanism.

According to all experimental data, we propose a mechanism for this formal (4+1) cycloaddition of cycloheptatrienes **1** and MCP in which gold(I) plays a triple role (Scheme 6). In the first catalytic cycle, η^2^-MCP-gold(I) complex **I** undergoes ring expansion to form intermediate **II**, which gives η^2^-cyclobutene-gold(I) complex **III**. Associative ligand exchange with the 7-aryl-1,3,5-cycloheptatriene, followed by retro-Buchner reaction then leads to the highly reactive gold(I) carbene **2**,^7^ which reacts with cyclobutene **4** to form bicyclo[2.1.0]pentane-gold(I) complex **IV**. Cyclopropane opening by gold(I) forms the tertiary carbocation **V**, which leads to complex **VI** by a final 1,2-H shift. The cyclopropanation of **4** by **2**, followed by electrophilic cleavage probably follows a pathway similar to that occurring in the gas phase for the cyclopropanation/retro-cyclopropanation of enol ethers with gold(I) carbenes.^22^ Formation of cyclopentenes from bicyclo[2.1.0]pentanes, the presumed intermediates of these reactions, has been mechanistically examined in a few cases using Rh^I^, Zn^II^, and other catalysts.^23^, ^24^ Formation of regioisomeric 3-alkyl-3-arylcyclopent-1-enes together with **5 n**–**p** in the reaction of alkyl-substituted MCP can be explained by the competitive migration of the aryl group in intermediates **V**.

**Scheme 6:**
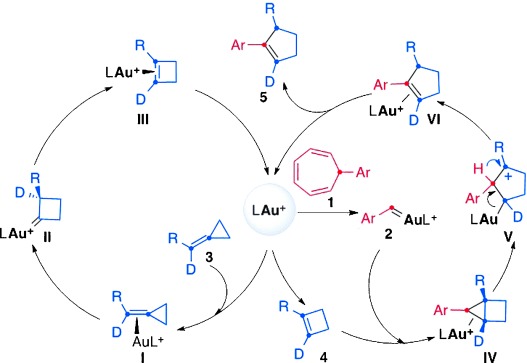
Proposed mechanism for the formal (4+1) cycloaddition.

In summary, we have developed a synthesis of substituted cyclopentenes by a formal (4+1) cycloaddition from methylenecyclopropanes or cyclobutenes with gold(I) carbenes generated under catalytic conditions by retro-Buchner reaction of 1,3,5-cycloheptatrienes or by other methods. Further work on the application of this cycloaddition in synthesis is underway.
